# Ultrasound-Induced Acoustic Cavitation Takes Centre Stage to Enhance Drug Delivery and the Activation of the Immune System

**DOI:** 10.3390/pharmaceutics17111430

**Published:** 2025-11-05

**Authors:** Petros Mouratidis, Ian Rivens, Gail ter Haar

**Affiliations:** Division of Radiotherapy and Imaging, The Institute of Cancer Research, London SM2 5NG, UK

## 1. Introduction

Ultrasound therapy has emerged as a powerful tool for the non-invasive treatment of indications ranging from Parkinson’s disease and essential tremor to malignant solid tumours. The 2025 State of the Field Report published by the Focused Ultrasound Foundation (https://cdn.fusfoundation.org/2025/07/31085318/FUSF-State-of-the-Field-2025_July-31.pdf accessed on 4 October 2025) shows that the total number of ultrasound therapy treatments has now exceeded 1 million, while US Federal funding for research in this area stands at just below USD 100M, up from approximately USD 10M 20 years ago. The sonication of tissues induces distinct physical effects such as acoustic cavitation, which harnesses bubble activity (“mechanical” effects) [1,2,3,4,5] and induces a temperature rise (“thermal” effects) [6,7]. These physical effects trigger biological responses, including the direct destruction of tissues, the permeabilization of biological barriers, improved gene and drug delivery, and the induction of an immune response [8,9,10].

This Special Issue, “Recent Advances in the Use of Ultrasound to Enhance Drug Delivery and Efficacy for the Treatment of Cancer”, has been proposed at a time when the interest in the “mechanical” effects of ultrasound therapy is surging, leading the technology towards widespread clinical adoption for the treatment of cancer. For example, there is a growing interest in “USMB”, the combination of low-pressure ultrasound (even below 1 MPa peak negative pressure) and microbubbles (gas-filled micron-sized bubbles that oscillate upon ultrasound stimulation and promote acoustic cavitation), which can be utilised to open the blood–brain barrier or to disrupt the tumour microenvironment, thus improving access to the interstitium of malignant lesions. The use of high-power histotripsy for the treatment of soft tissue tumours, such as those of the prostate, liver, kidney, and pancreas, via mechanical effects is also increasingly being explored [11,12]. This Special Issue therefore adjusts the focus from traditional hyperthermia-based approaches to improving drug delivery, to the use of adjuvant endogenously and exogenously injected bubbles to enhance the efficacy of chemotherapy, radiotherapy, and immunotherapy via acoustic cavitation.

## 2. Overview of Contents of This Special Issue

The use of ultrasound and contrast-enhanced imaging agents, including microbubbles and nanodroplets, has recently gained momentum in veterinary clinics as a cost-effective and rapid method for treating malignancies in terminally ill companion animals. The objective has been to increase the tolerability, safety, and anti-cancer effects of first-line chemotherapies. In their contribution, de Maar et al. have treated five feline subjects suffering from oral squamous cell carcinoma with low-power ultrasound and microbubbles to increase the blood perfusion in the tumours (1). Ultrasound treatments were delivered using pulse wave Doppler imaging (MI (Mechanical Index) = 0.3–0.4) for 15 s and were repeated five times at 5 s intervals per microbubble injection. The authors showed that tumour perfusion was increased in treated subjects, and, when combined with bleomycin, a modestly improved clinical response was produced, showing short-term stable disease progression for three out of the five subjects. All subjects survived longer than the historical records (46 to 147 days compared with 44 days).

Similar approaches have been investigated in models of the human version of the disease. In Almasri et al’s contribution (2), human prostate PC3 tumours grown in murine immune-deficient subjects were sonicated using peak negative pressures of approx. 0.5 MPa. The ultrasound and microbubble treatments were combined with docetaxel (5 mg/kg) and were followed immediately with external beam radiation (8 Gy in a single fraction). The triple treatment enhanced the killing of the cancer cells and the induction of apoptosis compared with all other single and dual treatments and eliminated the tumours. The anti-tumour effects were evident for at least 40 days after treatment, at which point all subjects in the other treatment cohorts had died. This study provided further proof-of-concept evidence that ultrasound and microbubbles enhance the effects of traditional oncologic interventions such as radiotherapy and chemotherapy.

The use of ultrasound and microbubbles to open the blood–brain barrier to enhance drug delivery is an intensely active research area. Nanoscale droplets (nanodroplets), which are smaller than microbubbles, can also cavitate upon ultrasound excitation. Their application in the permeabilization of endothelial barriers has been investigated by Vlatakis et al. (3). In this study, the authors characterised, in vitro, the effect of nanodroplets on endothelial monolayers that could mimic those of the blood–brain barrier. The nanodroplets were emulsions of different perfluorocarbon cores, and their physicochemical properties were investigated. The authors demonstrated nanodroplet cavitation at 0.66 MPa peak negative pressure ultrasound excitation using a high-speed camera and went on to show how this can compromise the integrity of the endothelial monolayers, demonstrated as an increase in sodium fluorescein permeability, in a more controlled way than achieved when they used microbubbles.

Yamaguchi et al. investigated the use of cavitation-inducing low-power ultrasound and nanoscale bubbles in an in vivo model of human lung squamous cell carcinoma grown in murine subjects (4). The objective was to enhance the delivery of plasmid-based CRISPR targeting p63 and SOX2 for degradation. In this study, nanobubbles inside the tumours were imaged using a wideband 12 MHz transducer, and ultrasound excitation was performed (experimental conditions: 1 MHz, 50% duty cycle, 2.0 W/cm^2^, and 2 min). The combination of ultrasound and nanobubbles induced transient membrane permeabilization, which increased gene delivery in the tumours by at least six-fold compared with the treatments without ultrasound and nanobubbles. When CRISPR against p63 and the ultrasound nanobubble treatments were combined, this resulted in significant tumour growth control of approximately 50% compared with the treatments without ultrasound. No adverse effects were seen on the health of the murine subjects.

Acoustic cavitation can be induced, not only by using exogenously injected contrast-enhancing agents, but also by taking advantage of ultrasound’s effects on pre-existing gas nuclei in the tissue. In this case, upon high-pressure ultrasound excitation, these gas nuclei grow into oscillating bubbles which collapse violently and release considerable amounts of energy. These treatments, known as histotripsy, directly destroy the tissue mechanically, without heating, and can induce an immune response. In the contribution from Mouratidis et al. (5), a well-known immune “cold” tumour model, pancreatic ductal adenocarcinoma, was used. The authors used multi-omics to show that the pancreatic tumour microenvironment consisted of T cells that expressed exhaustion markers and immune checkpoints, and neutrophils that expressed pro-tumour growth anti-immune markers. When the tumours were treated with cavitation-inducing histotripsy (with peak negative pressures of 17–19 MPa) combined with an oncolytic reovirus, a greater innate immune response was induced compared with either treatment alone or to sham-exposed tumours. To counteract the ablative effects of the treatments, the tumours attracted neutrophils that were programmed to express anti-immune pro-tumour growth genes. These neutrophils represent a new treatment target that could help improve pancreatic tumour growth control.

## 3. Future Perspectives

The Guest Editors believe that coupling ultrasound-induced acoustic cavitation with advanced drug delivery systems, such as those highlighted in this Special Issue, has the potential to show real human and veterinary clinical benefits. The use of AI, improved imaging techniques, improved transducer designs, and the standardisation of ultrasound exposure parameters among different manufacturers and research and clinical centres should overcome the current limitations in targeting deep-seated tumours with pulsed high-pressure ultrasound exposures, and could enable the most precise, safe, and rapid treatment for patients at an early disease stage. The potential of ultrasound to stimulate the immune system of patients to fight their own disease is still relatively unexplored. This is in part due to the immune system’s inherent biological complexity. The use of advanced techniques, such as spatial transcriptomics at the single-cell level, should allow a more thorough understanding of the immune processes underlying the response of tissues to ultrasound, and will provide information about the best immunotherapies that could be combined with ultrasound. This is an exciting era for ultrasound therapy research, and the growing number of clinical applications for ultrasound will shape future treatments of both humans and of our cherished companion animals.

## Abbreviations

The following abbreviations are used in this manuscript:

MI	Mechanical Index
USMB	Low-Pressure Ultrasound and Microbubbles
